# Inflammaging and Sarcopenia as Interconnected Hallmarks of Aging: Integrative Roles of Bioactive Compounds and Lifestyle Interventions

**DOI:** 10.3390/nu18121920

**Published:** 2026-06-13

**Authors:** Dorottya Nyáry, Mónika Fekete, Andrea Lehoczki, Vince Fazekas-Pongor, Ágnes Lipécz, Tamás Csípő, Dávid Major, Anna Péterfi, Boglárka Csík, Virág Zábó, Attila Matiscsák, János Tamás Varga

**Affiliations:** 1 Institute of Preventive Medicine and Public Health, Faculty of Medicine, Semmelweis University, 1085 Budapest, Hungary; nyary.dorottya@stud.semmelweis.hu (D.N.); fekete.monika@semmelweis.hu (M.F.); ceglediandi@freemail.hu (A.L.); pongor.vince@semmelweis.hu (V.F.-P.); lipecz.agnes@semmelweis.hu (Á.L.); csipo.tamas@semmelweis.hu (T.C.); major.david@semmelweis.hu (D.M.); peterfi.anna@semmelweis.hu (A.P.); csik.boglarka@semmelweis.hu (B.C.); zabo.virag@semmelweis.hu (V.Z.); 2 Health Sciences Division, Doctoral College, Semmelweis University, 1085 Budapest, Hungary; 3 Fodor Center for Prevention and Healthy Aging, Semmelweis University, 1085 Budapest, Hungary; 4 S-CAPE Cognitive and Health Prevention Research Group, Faculty of Health Sciences, Semmelweis University, 1088 Budapest, Hungary; matiscsak.attila@semmelweis.hu; 5 Department of Social Sciences, Faculty of Health Sciences, Semmelweis University, 1088 Budapest, Hungary; 6 Department of Pulmonology, Semmelweis University, 1083 Budapest, Hungary

**Keywords:** inflammaging, sarcopenia, bioactive compounds, lifestyle interventions, physical activity, oxidative stress, mitochondrial dysfunction, NF-κB, AMPK, mTOR, hormesis, geroscience, healthy aging

## Abstract

**Background/Objectives:** Age-related functional decline is increasingly linked to chronic low-grade inflammation (inflammaging) and sarcopenia, two interconnected processes contributing to frailty, metabolic dysregulation, and impaired physical function. These conditions share several underlying mechanisms, including immune dysregulation, mitochondrial dysfunction, oxidative stress, and impaired anabolic signaling. This narrative review critically evaluated the mechanistic and translational interactions between natural bioactive compounds and lifestyle interventions in modulating inflammaging and sarcopenia. **Methods:** Evidence from molecular, experimental, epidemiological, and clinical studies was synthesized to examine the effects of bioactive compounds—including polyphenols, flavonoids, carotenoids, and omega-3 fatty acids—as well as physical activity and dietary patterns. Particular emphasis was placed on inflammatory regulation, redox homeostasis, mitochondrial adaptation, and muscle metabolism, including NF-κB, AMPK–mTOR, and Nrf2 signaling pathways. **Results:** Observational studies and randomized controlled trials generally indicate that anti-inflammatory dietary patterns and regular physical activity are associated with improved muscle strength, physical performance, and inflammatory status in older adults. Mechanistically, nutritional bioactives and exercise appear to converge on several pathways involved in mitochondrial function, oxidative stress, anabolic signaling, and immune activation. Emerging evidence suggests potential convergence and interaction of biological pathways affected by nutritional and lifestyle interventions; however, formal evidence demonstrating true synergistic effects in humans remains limited. Nevertheless, substantial heterogeneity persists regarding intervention protocols, dosage strategies, bioavailability, and long-term clinical outcomes. **Conclusions:** Natural bioactive compounds and lifestyle-based interventions represent promising approaches for targeting biological processes implicated in inflammaging and sarcopenia. By integrating current evidence within a hormesis-oriented geroscience framework, this review highlights the importance of adaptive redox regulation, metabolic resilience, and evidence-based lifestyle strategies in healthy aging. Future well-designed longitudinal and intervention studies are needed to clarify the clinical relevance of these interactions and optimize translational implementation.

## 1. Introduction

The growing burden of age-related functional decline and multimorbidity has increasingly shifted scientific attention toward the biological mechanisms driving not only disease development but also the progressive loss of physical function and independence [1]. Consequently, contemporary concepts of healthy aging extend beyond the mere absence of disease and increasingly emphasize the preservation of functional capacity, resilience, and quality of life [2]. Among the major biological hallmarks contributing to this process are chronic low-grade inflammation (“inflammaging”) and sarcopenia, two closely interconnected conditions linked through bidirectional pathophysiological mechanisms [3].

Age-associated immune dysregulation promotes a persistent pro-inflammatory milieu that accelerates muscle protein catabolism, impairs anabolic signaling, and disrupts tissue homeostasis. Simultaneously, progressive skeletal muscle loss further amplifies systemic inflammation and metabolic dysfunction, thereby establishing a self-perpetuating pathological cycle [4]. In parallel, oxidative stress, mitochondrial dysfunction, and impaired cellular stress responses contribute to altered muscle metabolism, reduced physical performance, frailty, and increased vulnerability to adverse clinical outcomes. Importantly, these processes extend beyond skeletal muscle and reflect broader maladaptive alterations involving metabolic, neuromuscular, immune, and cognitive pathways [5].

Given the limited efficacy of current pharmacological approaches and the high prevalence of polypharmacy among older adults, increasing interest has emerged in non-pharmacological, multi-target interventions capable of modulating fundamental aging-related pathways [6]. In this context, dietary patterns, natural bioactive compounds, and lifestyle-related factors—particularly regular physical activity—have demonstrated the potential to influence several molecular mechanisms implicated in inflammaging and sarcopenia, including nuclear factor kappa B (NF-κB) signaling, the adenosine monophosphate-activated protein kinase (AMPK)–mechanistic target of rapamycin (mTOR) axis, redox homeostasis, mitochondrial adaptation, and cellular stress-response pathways [7,8,9].

Despite accumulating evidence supporting the beneficial effects of these interventions, most studies have evaluated nutritional compounds and lifestyle-related factors in isolation, providing limited insight into their biological interactions and potential complementary or convergent effects [10]. Moreover, the distinction between additive effects, biological pathway convergence, and true mechanistic synergy remains insufficiently explored, particularly in the context of adaptive hormetic responses and redox-sensitive signaling networks involved in healthy aging.

Accordingly, this narrative review provides a translational perspective on the role of natural bioactive compounds and lifestyle-based interventions in the regulation of inflammaging and sarcopenia. Particular emphasis is placed on shared molecular pathways, redox-sensitive adaptive responses, mitochondrial resilience, and evidence derived from human studies. By integrating mechanistic insights with clinical observations, this review aims to highlight multi-component strategies that may target key biological processes underlying age-related functional decline.

While previous reviews have examined nutritional interventions, physical activity, or specific classes of bioactive compounds individually, comparatively few have explored inflammaging and sarcopenia within an integrated geroscience framework. The present review focuses on the convergence of lifestyle-related factors and nutritional bioactives across interconnected molecular pathways, including NF-κB, AMPK–mTOR, Nrf2, and mitochondrial signaling networks. Particular attention is given to adaptive stress responses and hormetic regulation as potential mechanistic interfaces linking inflammation control, metabolic resilience, and the preservation of muscle function during aging. Rather than assuming synergistic effects, the review critically examines the extent to which current mechanistic and clinical evidence supports biologically convergent, complementary, or potentially interactive actions of these interventions. In doing so, it seeks to provide a balanced and translationally relevant synthesis of current knowledge at the intersection of inflammaging, sarcopenia, nutrition, and lifestyle medicine.

## 2. Methods

### 2.1. Literature Search Strategy

A comprehensive literature search was conducted using the PubMed/MEDLINE, Scopus, and Web of Science databases. The final search was performed in March 2026. Search strategies combined controlled vocabulary terms and keyword-based queries related to inflammaging, sarcopenia, bioactive compounds, nutrition, physical activity, exercise, oxidative stress, mitochondrial dysfunction, healthy aging, and lifestyle interventions. Representative search terms included “inflammaging”, “chronic low-grade inflammation”, “sarcopenia”, “bioactive compounds”, “polyphenols”, “omega-3 fatty acids”, “physical activity”, “exercise”, and “healthy aging”, combined using Boolean operators (AND, OR). Representative search strings included (“inflammaging” OR “chronic low-grade inflammation”) AND sarcopenia AND exercise in PubMed/MEDLINE; inflammaging AND sarcopenia AND bioactive compounds in Scopus; and inflammaging AND healthy aging AND lifestyle interventions in Web of Science. Detailed search strategies for each database are provided in Supplementary Table S1.

The search primarily focused on studies published between 2010 and 2026. Earlier landmark publications were additionally included when considered essential for establishing the conceptual and biological framework of the field. Only articles published in English were considered. Reference lists of relevant reviews and key original articles were also manually screened to identify additional eligible publications.

A structured screening process was applied to assess relevance. Approximately 4800 records were identified through database searching, with an additional 75 records identified through manual screening of reference lists. After duplicate removal, approximately 3650 records underwent title and abstract screening. Subsequently, 485 full-text articles were assessed for eligibility, of which 228 studies were considered sufficiently relevant and were included in the final narrative evidence synthesis (Figure S1). Studies were prioritized based on methodological quality, translational relevance, recency, and direct relevance to the interaction between inflammaging, sarcopenia, bioactive compounds, and lifestyle interventions.

Studies directly addressing the relationships among inflammaging, sarcopenia, bioactive compounds, and lifestyle interventions were subsequently included in the final evidence synthesis. Given the narrative nature of the review, the objective was not to identify all available publications exhaustively but rather to capture the most relevant mechanistic, translational, and clinical evidence related to inflammaging, sarcopenia, bioactive compounds, and lifestyle interventions.

### 2.2. Inclusion and Exclusion Criteria

Priority was given to human studies, randomized controlled trials, systematic reviews, meta-analyses, and mechanistic investigations addressing the interactions among inflammaging, sarcopenia, bioactive compounds, and lifestyle-related interventions. Particular emphasis was placed on studies evaluating multi-component interventions and potential interactions between nutritional and exercise-related factors.

Potential interactions between nutritional and lifestyle interventions were interpreted within a biological framework involving shared signaling pathways, adaptive stress responses, and coordinated metabolic regulation. Formal synergistic effects were considered only when supported by direct experimental or clinical evidence. Studies were excluded if they were not directly relevant to the topic or relied exclusively on in vitro findings without clear translational or clinical relevance.

### 2.3. Data Extraction and Evidence Synthesis

Key study characteristics—including study design, population characteristics, interventions, molecular targets, and clinical outcomes—were systematically evaluated. Evidence was synthesized into thematic domains encompassing molecular mechanisms, physiological adaptations, and functional outcomes. Particular emphasis was placed on pathways implicated in both inflammaging and sarcopenia, including NF-κB signaling, the AMPK–mTOR axis, redox regulation, mitochondrial function, and cellular stress-response pathways. Evidence derived from observational studies, randomized controlled trials, systematic reviews, meta-analyses, and experimental investigations was considered separately whenever possible to facilitate interpretation of the strength and translational relevance of the available evidence.

### 2.4. Evidence Appraisal and Methodological Considerations

The interpretation of human studies considered study design, sample size, methodological quality, and potential sources of bias. Randomized controlled trials and meta-analyses were weighted more heavily during evidence synthesis, whereas experimental and animal studies were primarily used to support biological plausibility and mechanistic interpretation.

Given the substantial heterogeneity across study populations, intervention protocols, outcome measures, and methodological approaches, the objective of this review was not to perform a quantitative pooled analysis. Instead, a structured narrative approach was adopted to provide a translational overview consistent with contemporary geroscience concepts and multi-target models of healthy aging. Because formal risk-of-bias assessment tools and GRADE methodology were not applied, evidence appraisal should be interpreted as a structured narrative evaluation rather than a quantitative evidence-ranking process.

## 3. Pathophysiological Basis of Inflammaging and Sarcopenia

### 3.1. Inflammaging

Chronic low-grade inflammation associated with aging, commonly termed inflammaging, is increasingly recognized as a central hallmark of biological aging [11]. Although no universally accepted clinical diagnostic criteria currently exist for inflammaging, the condition is generally characterized by persistent, low-grade systemic inflammation in the absence of overt infection [11]. In research settings, inflammaging is commonly assessed using circulating inflammatory biomarkers, including C-reactive protein (CRP), interleukin-6 (IL-6), tumor necrosis factor alpha (TNF-α), and, less frequently, interleukin-1β (IL-1β). Elevated concentrations of these biomarkers have been consistently associated with frailty, sarcopenia, disability, multimorbidity, and increased mortality risk in older adults. Recent clinical evidence further suggests that poor nutritional status in older adults is associated with increased oxidative stress, altered adipokine profiles, and markers of chronic inflammation, supporting the close interaction among malnutrition, inflammaging, and age-related functional decline [11,12]. Among these biomarkers, IL-6 and CRP are the most frequently used indicators of inflammaging in epidemiological and clinical studies owing to their robust associations with adverse aging-related outcomes [12]. Although no universally accepted biomarker cut-offs have been established for inflammaging, epidemiological studies frequently use elevated CRP (>3 mg/L) and higher circulating IL-6 concentrations as indicators of increased age-related inflammatory burden. However, these biomarkers should be interpreted within the broader clinical and biological context rather than as diagnostic criteria for inflammaging. Importantly, inflammaging is increasingly viewed as a multidimensional biological process involving immune dysregulation, cellular senescence, mitochondrial dysfunction, and impaired resolution of inflammation rather than a single biomarker-defined condition [11,12]. This process emerges from the cumulative interaction of these biological alterations and the progressive accumulation of molecular damage during aging [12]. Age-related immunosenescence contributes substantially to this phenomenon by impairing adaptive immune responses while simultaneously promoting persistent activation of the innate immune system, thereby favoring a chronic pro-inflammatory state [13].

Cellular senescence represents one of the principal drivers of inflammaging. Senescent cells acquire a characteristic senescence-associated secretory phenotype (SASP), characterized by the sustained release of pro-inflammatory cytokines, chemokines, growth factors, and matrix-remodeling enzymes [14]. Beyond local tissue effects, SASP-associated mediators contribute to systemic inflammatory signaling, reinforce neighboring cellular senescence, and disrupt tissue homeostasis across multiple organ systems [15].

Among the major inflammatory mediators implicated in inflammaging, interleukin-6 (IL-6) and tumor necrosis factor alpha (TNF-α) play particularly prominent roles [16]. These cytokines activate NF-κB, a central regulator of inflammatory gene transcription and cellular stress responses [17]. Persistent NF-κB activation not only sustains chronic inflammation but also promotes tissue catabolism, anabolic resistance, and metabolic dysfunction, thereby providing a mechanistic link between inflammaging and sarcopenia [7,18]. In parallel, activation of the NLR family pyrin domain containing 3 (NLRP3) inflammasome further amplifies inflammatory signaling through increased production of interleukin-1 beta (IL-1β) and interleukin-18 (IL-18) [19].

Oxidative stress and mitochondrial dysfunction constitute additional amplifiers of inflammaging [20,21,22,23]. Increased generation of reactive oxygen species (ROS), together with declining endogenous antioxidant defenses, contributes not only to cumulative molecular damage but also to redox-sensitive inflammatory signaling [24,25,26,27]. Importantly, dysfunctional mitochondria release danger-associated molecular patterns (DAMPs), including mitochondrial DNA (mtDNA), which further activate innate immune pathways and perpetuate chronic inflammatory activity [28]. Together, these mechanisms indicate that inflammaging represents a multifactorial process involving immune, metabolic, and mitochondrial dysfunction rather than an isolated inflammatory state. This pathophysiological framework provides a mechanistic basis for understanding the development and progression of sarcopenia and other age-related functional disorders.

### 3.2. Sarcopenia

Sarcopenia is a multifactorial and progressive skeletal muscle disorder characterized by the loss of muscle mass, strength, and physical function, driven by disturbances in anabolic signaling, mitochondrial homeostasis, neuromuscular integrity, and inflammatory regulation [19,28]. According to the revised European Working Group on Sarcopenia in Older People (EWGSOP2), low muscle strength is considered the primary indicator of probable sarcopenia, whereas the diagnosis is confirmed by the presence of reduced muscle quantity or quality. Sarcopenia is considered severe when low muscle strength, low muscle quantity or quality, and impaired physical performance coexist. Recommended assessment tools include handgrip strength and the chair stand test for muscle strength, dual-energy X-ray absorptiometry (DXA) and bioelectrical impedance analysis (BIA) for muscle quantity, and gait speed or the Short Physical Performance Battery (SPPB) for evaluation of physical performance. This operational definition emphasizes that muscle strength and function are more clinically relevant predictors of adverse outcomes than muscle mass alone [29,30]. Importantly, intervention effects on muscle mass, muscle strength, and physical performance should be interpreted separately, as these outcomes do not necessarily improve in parallel. Several nutritional and lifestyle interventions demonstrate stronger effects on muscle strength and functional performance than on muscle mass itself. Central to its pathophysiology is anabolic resistance, defined as the impaired responsiveness of skeletal muscle to anabolic stimuli such as dietary protein intake and physical activity [31]. This phenomenon is closely associated with reduced activation of the mTOR pathway, impaired insulin signaling, and diminished muscle protein synthesis (MPS) [32].

In parallel, catabolic pathways become increasingly dominant during aging, particularly through activation of the ubiquitin–proteasome system and autophagy-related degradation pathways [33]. Chronic exposure to pro-inflammatory cytokines, including TNF-α and IL-6, further accelerates muscle protein breakdown (MPB) while simultaneously suppressing anabolic signaling and impairing regenerative capacity [34]. These alterations establish a direct biological link between inflammaging and progressive skeletal muscle deterioration.

Age-related remodeling of the neuromuscular system also contributes substantially to declining muscle strength and physical performance [25,35,36]. Progressive motor neuron loss, muscle fiber denervation, and structural impairments of the neuromuscular junction collectively compromise motor unit recruitment, muscle activation, and coordination [37]. Importantly, these neuromuscular alterations may precede measurable declines in muscle mass, underscoring the multifactorial nature of sarcopenia beyond simple muscle atrophy.

Mitochondrial dysfunction represents another critical feature of sarcopenia and contributes directly to impaired muscular energy metabolism and reduced adaptive capacity [25,38,39]. Increased ROS production, impaired oxidative phosphorylation, reduced adenosine triphosphate (ATP) synthesis, and defective mitophagy collectively disrupt cellular bioenergetics and compromise skeletal muscle regeneration [40]. These processes are further influenced by endocrine alterations, insulin resistance, physical inactivity, and age-related changes in the gut microbiome, all of which contribute to disease progression [41]. Collectively, sarcopenia should be viewed not merely as a disorder of muscle loss, but as a complex age-related condition involving metabolic, inflammatory, mitochondrial, and neuromuscular dysfunction. This perspective provides a strong rationale for integrated lifestyle-based interventions targeting multiple biological pathways simultaneously.

### 3.3. Interaction Between Inflammaging and Sarcopenia

The relationship between inflammaging and sarcopenia is now widely recognized as a self-reinforcing biological axis in which inflammatory, metabolic, and musculoskeletal alterations interact through multiple overlapping mechanisms [42]. Rather than representing independent age-related conditions, inflammaging and sarcopenia evolve through reciprocal dysregulation of cellular stress responses, anabolic signaling, immune activation, and energy metabolism.

Chronic low-grade inflammation suppresses anabolic pathways involved in skeletal muscle maintenance, particularly through inhibition of the mTOR signaling axis, while simultaneously promoting activation of catabolic systems, including the ubiquitin–proteasome pathway and autophagy-related degradation mechanisms [43]. In parallel, pro-inflammatory mediators impair insulin signaling and glucose utilization, thereby reducing metabolic flexibility and energy availability within skeletal muscle tissue [44]. Collectively, these alterations contribute to anabolic resistance, mitochondrial dysfunction, and progressive muscle catabolism.

Conversely, age-related skeletal muscle loss and declining physical activity further exacerbate systemic metabolic dysfunction. Reduced muscle mass diminishes glucose disposal capacity and promotes adipose tissue accumulation, particularly within visceral fat depots [45]. Importantly, visceral adipose tissue functions as a metabolically active endocrine organ characterized by increased secretion of pro-inflammatory cytokines and adipokines, thereby amplifying chronic systemic inflammation [46].

Together, these mechanisms establish a feed-forward pathological cycle linking chronic inflammation, metabolic dysfunction, and skeletal muscle deterioration. Clinically, this inflammatory–musculoskeletal axis contributes to declining physical performance, frailty, disability, loss of independence, and reduced quality of life in older adults [46,47]. These interactions support the rationale for integrated interventions targeting multiple aging-related pathways.

## 4. Natural Bioactive Compounds in the Regulation of Inflammaging and Sarcopenia

### 4.1. Polyphenols and Flavonoids

Polyphenols and flavonoids are among the most extensively investigated plant-derived bioactive compounds and have attracted considerable interest in geroscience research because of their anti-inflammatory, antioxidant, and metabolic regulatory properties [48]. Rather than acting through single molecular targets, these compounds influence multiple pathways involved in cellular stress responses, mitochondrial adaptation, immune regulation, and metabolic homeostasis, thereby modulating processes implicated in both inflammaging and sarcopenia [48,49].

Experimental and clinical evidence suggests that polyphenols may attenuate chronic low-grade inflammation through suppression of NF-κB signaling, modulation of ROS-dependent pathways, and activation of AMPK- and SIRT1-mediated adaptive responses [50,51,52,53]. In parallel, several flavonoids have demonstrated the potential to improve mitochondrial function, redox balance, and cellular adaptability under conditions of age-related metabolic stress.

Importantly, the biological effects of polyphenols appear to depend not only on dosage and bioavailability, but also on the broader metabolic and lifestyle context in which they are administered or consumed. Emerging evidence suggests that certain bioactive compounds may exert hormetic effects, whereby mild cellular stress activates adaptive pathways associated with enhanced stress resistance and metabolic flexibility. However, despite strong mechanistic and preclinical evidence, translation into clinically meaningful improvements in muscle mass and functional outcomes remains inconsistent across human studies.

Table 1 summarizes the principal molecular pathways, physiological effects, and current levels of evidence associated with major classes of bioactive compounds implicated in the regulation of inflammaging and sarcopenia.

#### 4.1.1. Mechanistic Effects

Experimental evidence indicates that polyphenols modulate multiple signaling pathways implicated in inflammaging and age-related metabolic dysfunction [68]. Among the most consistently reported mechanisms is suppression of NF-κB signaling, leading to reduced transcription of pro-inflammatory cytokines and attenuation of chronic inflammatory activity. In parallel, modulation of mitogen-activated protein kinase (MAPK) pathways contributes to the regulation of cellular stress responses, inflammatory signaling, and redox-sensitive adaptation [69].

A central aspect of polyphenol activity involves the regulation of mitochondrial function and cellular energy metabolism. Experimental evidence indicates that several polyphenols activate AMPK and SIRT1, two major regulators of mitochondrial biogenesis, oxidative metabolism, autophagy, and stress resistance [70]. Specific polyphenols such as luteolin have also been reported to modulate AMPK-, Nrf2-, and NF-κB-related signaling pathways, suggesting potential roles in the regulation of oxidative stress responses, inflammation, and metabolic homeostasis [71]. Through these mechanisms, polyphenols may improve oxidative phosphorylation efficiency, enhance mitochondrial quality control, and promote mitophagy-mediated clearance of dysfunctional mitochondria. These effects are particularly relevant in inflammaging, where mitochondrial dysfunction and impaired redox homeostasis contribute directly to persistent inflammatory signaling and metabolic decline [69,70,71].

Polyphenols may additionally influence epigenetic regulation, including histone modification, DNA methylation, and microRNA expression, thereby affecting long-term patterns of gene expression and cellular adaptation [72,73]. Collectively, these mechanisms illustrate the complex molecular interactions through which polyphenols may modulate biological aging and age-related functional decline [73]. Nevertheless, despite substantial mechanistic and preclinical evidence, the translation of these molecular effects into clinically meaningful human outcomes remains incompletely understood. Variability in bioavailability, dosage, intervention duration, and study design continues to limit direct extrapolation from experimental findings to clinical practice [73].

#### 4.1.2. Preclinical Evidence

Preclinical studies consistently demonstrate that polyphenol supplementation attenuates inflammatory signaling, reduces oxidative stress, and improves mitochondrial function in aging-related experimental models [74]. Several experimental studies have additionally reported improvements in muscle metabolism, mitochondrial biogenesis, autophagic activity, and skeletal muscle performance, supporting a potential role for polyphenols in modulating both inflammaging and sarcopenia [19,75,76,77].

Mechanistically, these effects appear to involve modulation of NF-κB-, AMPK-, SIRT1-, and other redox-sensitive signaling pathways, together with improvements in mitochondrial quality control and cellular stress adaptation. Experimental findings further suggest that certain polyphenols may induce hormetic responses, whereby mild cellular stress activates adaptive pathways associated with enhanced metabolic flexibility and stress resilience.

Nevertheless, these beneficial effects are often dose-dependent, and the concentrations used in experimental settings frequently exceed levels achievable through habitual dietary intake in humans [78,79]. Differences in bioavailability, metabolism, and tissue distribution further complicate the translation of preclinical findings into clinical practice. Accordingly, although experimental evidence strongly supports the mechanistic rationale for polyphenol-based interventions, their translational relevance in humans remains incompletely established.

#### 4.1.3. Human Evidence

Observational evidence suggests that polyphenol-rich dietary patterns, particularly the Mediterranean diet, are associated with lower systemic inflammatory burden and improved physical function in older adults [80,81]. Randomized controlled trials further suggest that specific flavonoids, including cocoa flavanols and green tea catechins, may improve endothelial function, vascular responsiveness, and selected inflammatory biomarkers [82,83].

However, evidence regarding direct effects on skeletal muscle mass, strength, and clinically relevant sarcopenia outcomes remains heterogeneous and inconclusive. Considerable variability across studies in intervention duration, dosage, compound composition, bioavailability, participant characteristics, and outcome assessment methods limits comparability and weakens clinical interpretation. Importantly, many reported benefits appear to arise primarily from indirect modulation of inflammatory, vascular, and metabolic pathways rather than from pronounced anabolic effects on skeletal muscle itself. This distinction is particularly relevant when evaluating the translational potential of polyphenols for the prevention and management of sarcopenia.

Overall, current human evidence suggests a modulatory role for polyphenols in pathways related to inflammaging, oxidative stress, endothelial dysfunction, and metabolic regulation. Effects on inflammatory markers appear more consistent than those observed for muscle-related outcomes. Nevertheless, their capacity to induce clinically meaningful improvements in muscle mass, muscle strength, and long-term functional outcomes remains insufficiently established and warrants further large-scale, well-controlled clinical studies [74].

### 4.2. Carotenoids and Other Lipophilic Bioactive Compounds

Carotenoids, including β-carotene, lutein, and lycopene, are lipid-soluble bioactive compounds with established antioxidant, cytoprotective, and immunomodulatory properties [84]. Increasing evidence suggests that their biological activity extends beyond direct radical scavenging and involves broader regulation of inflammatory, metabolic, and redox-sensitive signaling pathways implicated in biological aging [85].

The membrane-stabilizing properties of carotenoids appear particularly relevant in aging tissues, where increased lipid peroxidation, mitochondrial dysfunction, and impaired membrane integrity contribute substantially to cellular stress and metabolic decline [86]. By modulating membrane lipid composition and the surrounding redox microenvironment, carotenoids may influence membrane-associated signaling pathways involved in inflammation, mitochondrial adaptation, and cellular stress responses [87].

At the molecular level, carotenoids can attenuate ROS-mediated oxidative stress, thereby modulating one of the central drivers of inflammaging. Importantly, their biological effects are increasingly recognized as regulatory rather than purely antioxidant in nature [88]. Through modulation of cellular redox status, carotenoids influence inflammatory pathways such as NF-κB signaling and may additionally contribute to suppression of inflammasome activation and chronic innate immune stimulation [7].

Carotenoids also exhibit immunomodulatory properties that may support immune homeostasis during aging by influencing the balance between pro-inflammatory and anti-inflammatory responses [89]. This effect is particularly relevant in inflammaging, where persistent immune dysregulation contributes to systemic metabolic and functional decline [90].

Observational studies consistently report associations between higher carotenoid intake and lower inflammatory biomarker levels, improved antioxidant status, and better physical performance in older adults [91]. Nevertheless, most available human evidence remains observational, limiting causal interpretation and translational applicability.

An additional challenge involves the marked variability in carotenoid bioavailability, which is influenced by dietary matrix composition, fat intake, interindividual metabolic differences, and gut microbiome-related factors [92]. Such variability likely contributes to the heterogeneity observed across intervention studies and complicates the development of standardized clinical recommendations. Current evidence supports a complementary modulatory role for carotenoids in pathways related to inflammaging, oxidative stress, and metabolic dysfunction. However, their direct impact on sarcopenia-related outcomes and long-term functional trajectories remains insufficiently established and warrants further mechanistically informed human intervention studies.

### 4.3. Omega-3 Fatty Acids and Lipid-Derived Bioactive Compounds

Omega-3 polyunsaturated fatty acids (PUFAs), particularly eicosapentaenoic acid (EPA) and docosahexaenoic acid (DHA), represent among the most extensively investigated and clinically relevant nutritional modulators of inflammaging and sarcopenia [93]. Their biological effects extend beyond suppression of pro-inflammatory signaling and include active regulation of inflammation-resolution pathways [94,95,96,97,98].

Central to this process is the biosynthesis of specialized pro-resolving mediators (SPMs), including resolvins, protectins, and maresins, which not only attenuate inflammatory activity but also actively promote resolution of inflammation, restoration of tissue homeostasis, and recovery of cellular function [94,99,100]. This distinction is particularly important in inflammaging, where chronic inflammation is characterized less by excessive acute immune activation than by impaired resolution and persistent low-grade inflammatory signaling.

At the molecular level, omega-3 fatty acids modulate multiple pathways involved in inflammatory regulation, metabolic homeostasis, and skeletal muscle adaptation. EPA and DHA suppress activation of NF-κB while simultaneously influencing peroxisome proliferator-activated receptor (PPAR)-mediated transcriptional pathways, thereby coordinating inflammatory and metabolic responses [101,102]. In addition, incorporation of omega-3 fatty acids into cellular membranes alters membrane fluidity and receptor-associated signaling, contributing to improved insulin sensitivity and metabolic responsiveness [93,94,103].

From a skeletal muscle perspective, omega-3 fatty acids appear particularly relevant in the regulation of anabolic sensitivity. Experimental and clinical evidence suggests that EPA and DHA enhance responsiveness of the mTOR pathway to anabolic stimuli such as amino acids, insulin, and resistance exercise [104]. Through these mechanisms, omega-3 fatty acids may partially counteract anabolic resistance, a central hallmark of sarcopenia and age-related muscle dysfunction [56].

Evidence from randomized controlled trials and meta-analyses indicates that omega-3 supplementation consistently improves muscle strength and selected measures of physical performance in older adults [105]. By contrast, effects on muscle mass are generally smaller and less consistent across studies [94]. These findings suggest that the primary benefits of omega-3 fatty acids may relate more to improvements in muscle quality, neuromuscular efficiency, and functional performance than to substantial increases in skeletal muscle mass alone [106]. Furthermore, several studies have reported favorable effects on inflammatory biomarkers, supporting a potential role of omega-3 fatty acids in modulating inflammaging-related processes [55,56,57].

Nevertheless, considerable heterogeneity remains across clinical studies, likely reflecting differences in dosage, intervention duration, baseline nutritional status, habitual dietary patterns, physical activity levels, and interindividual metabolic responsiveness [107]. Bioavailability and interactions with broader lifestyle factors may further influence therapeutic efficacy and contribute to variability in clinical outcomes [108].

Omega-3 fatty acids appear to represent promising nutritional modulators of inflammaging and sarcopenia, particularly within integrated lifestyle-based intervention strategies. Their combined anti-inflammatory, pro-resolving, metabolic, and neuromuscular effects position them as strong candidates for multi-target approaches aimed at preserving functional capacity during aging. Future research should focus on identifying responder phenotypes, optimizing dose–response relationships, and clarifying potential synergistic interactions with exercise and other dietary interventions.

### 4.4. Bioactive Peptides and Fermentation-Derived Components

Bioactive peptides and fermentation-derived compounds have emerged as increasingly relevant modulators of aging-related biological processes, particularly at the intersection of nutrition, gut microbiome dynamics, systemic inflammation, and skeletal muscle metabolism [109]. Although this field remains less established than research on polyphenols or omega-3 fatty acids, accumulating evidence suggests that these compounds may influence multiple pathways implicated in inflammaging and sarcopenia.

Bioactive peptides are typically generated through enzymatic hydrolysis or microbial fermentation of dietary proteins and exhibit diverse biological activities related to muscle metabolism, inflammatory regulation, oxidative stress responses, and immune modulation [110]. Experimental studies indicate that certain peptides may stimulate muscle protein synthesis, enhance anabolic signaling, and modulate pathways associated with mTOR activation and muscle regeneration [111]. Other peptide fractions appear capable of attenuating inflammatory signaling through regulation of pro-inflammatory cytokine production and redox-sensitive pathways.

Nevertheless, much of the current mechanistic evidence derives from preclinical and experimental models, whereas robust human intervention studies remain limited. In addition, the biological efficacy of bioactive peptides is strongly influenced by peptide structure, gastrointestinal stability, absorption kinetics, metabolism, and bioavailability, all of which contribute to substantial interindividual variability [112,113].

In parallel, fermentation-derived bioactive compounds may exert broader systemic effects through modulation of the gut microbiome and its metabolic outputs [114]. Fermented foods and microbiota-associated metabolites can influence microbial diversity, intestinal barrier integrity, and production of short-chain fatty acids (SCFAs), which are increasingly recognized as important regulators of immune homeostasis, inflammatory signaling, and metabolic function [115].

This concept has contributed to growing interest in the gut–muscle axis, a bidirectional network connecting intestinal microbial activity with skeletal muscle metabolism and functional aging [116]. Microbiome-derived metabolites may influence insulin sensitivity, mitochondrial function, inflammatory status, and anabolic responsiveness, thereby affecting muscle quality and physical performance through indirect metabolic and immunological mechanisms [117].

Despite considerable mechanistic interest, the current evidence base remains heterogeneous and translationally limited. Large-scale, long-term randomized controlled trials are still lacking, and clinically meaningful effects on sarcopenia-related outcomes have yet to be consistently demonstrated. Consequently, bioactive peptides and fermentation-derived compounds should presently be regarded as promising but still evolving components of multi-target nutritional strategies aimed at modulating inflammaging and age-related functional decline. To facilitate translational interpretation of the available evidence, representative characteristics of human studies evaluating major bioactive compounds, including study populations, formulations, dose ranges, intervention durations, and principal functional outcomes, are summarized in Supplementary Table S2.

Future research should prioritize mechanistically informed clinical studies capable of identifying responder populations, clarifying microbiome-dependent effects, and determining whether these interventions can produce durable improvements in muscle function, metabolic resilience, and healthy aging trajectories.

## 5. Role of Lifestyle Factors in the Regulation of Inflammaging and Sarcopenia

### 5.1. Physical Activity and Exercise Adaptations

Physical activity represents one of the most robust and consistently supported non-pharmacological interventions for modulating age-related inflammatory and metabolic dysfunction [118]. Its effects extend across multiple biological systems and involve coordinated regulation of inflammatory signaling, mitochondrial adaptation, proteostasis, metabolic flexibility, and skeletal muscle remodeling [118,119].

Regular exercise—particularly resistance training and multicomponent exercise interventions—has been shown to attenuate chronic low-grade inflammation through modulation of NF-κB signaling, suppression of NLR family pyrin domain containing 3 (NLRP3) inflammasome activation, and regulation of immune–metabolic pathways [120]. These adaptations contribute to reductions in circulating pro-inflammatory mediators and improvements in metabolic homeostasis. Importantly, the magnitude and direction of these responses depend strongly on exercise modality, intensity, training volume, recovery status, and baseline physiological condition [121,122].

Resistance training is widely regarded as one of the principal non-pharmacological approaches for the prevention and management of sarcopenia [123]. Mechanical loading activates mechanotransduction pathways converging on the mTOR signaling axis, thereby stimulating muscle protein synthesis while suppressing proteolytic and catabolic pathways [32]. In contrast, endurance-oriented exercise primarily promotes mitochondrial biogenesis, oxidative capacity, and metabolic adaptation through activation of AMPK and related energy-sensing pathways [124,125].

The endocrine function of skeletal muscle further highlights the systemic effects of exercise adaptation. Exercise-induced release of myokines, particularly IL-6, exerts context-dependent immunoregulatory effects that differ fundamentally from the chronic elevation of IL-6 observed during inflammaging [126]. Acute exercise-associated increases in IL-6 may promote anti-inflammatory signaling and metabolic adaptation, underscoring that inflammatory signaling can serve adaptive rather than exclusively pathological functions [127].

Exercise also enhances autophagy, mitophagy, and mitochondrial quality control, thereby improving proteostasis and cellular stress tolerance [128]. These mechanisms are particularly relevant during aging, when impaired clearance of dysfunctional proteins and mitochondria contributes to persistent inflammatory activation, metabolic decline, and reduced muscle function [129]. Overall, physical activity exerts broad regulatory effects on pathways implicated in both inflammaging and sarcopenia [130]. Rather than targeting isolated mechanisms, exercise induces coordinated adaptations across inflammatory, metabolic, neuromuscular, and mitochondrial pathways. Nevertheless, substantial interindividual variability exists in responsiveness to exercise interventions, highlighting the importance of personalized and precision-based exercise strategies in healthy aging.

### 5.2. Dietary Patterns and Energy Balance

Dietary patterns play a central role in the regulation of inflammaging and sarcopenia through their effects on inflammatory signaling, metabolic homeostasis, hormonal regulation, and skeletal muscle metabolism [131,132,133]. Increasing evidence suggests that overall dietary quality and long-term dietary behavior may exert greater biological relevance than isolated nutrient supplementation alone, particularly in the context of healthy aging.

Among currently studied dietary models, the Mediterranean diet represents one of the most consistently supported anti-inflammatory and cardiometabolic dietary patterns [77,134,135]. Importantly, its beneficial effects appear to arise from the combined action of multiple nutritional components—including polyphenols, unsaturated fatty acids, dietary fiber, vitamins, and other bioactive compounds—rather than from any single nutrient in isolation [52,98,100,136,137]. This nutritional profile has been associated with reductions in inflammatory biomarkers, improved endothelial and metabolic function, enhanced insulin sensitivity, and better preservation of physical performance in older adults [138].

Nevertheless, the available evidence demonstrates considerable heterogeneity, particularly across intervention studies. Variability in dietary adherence, baseline metabolic status, population characteristics, study duration, and outcome assessment methods complicates direct comparison between studies and limits the strength of causal inference [139].

Observational and mechanistic evidence suggests that plant-based dietary patterns may exert partially overlapping biological effects through modulation of inflammatory, metabolic, and redox-sensitive pathways. Importantly, these effects are influenced not only by nutrient composition but also by the structural and biochemical properties of the food matrix, which affect digestion, nutrient bioavailability, gut microbial metabolism, and downstream physiological responses [140]. Sustainable dietary patterns, including predominantly plant-based dietary approaches, may contribute to healthy aging when adequate intake of protein, vitamin B12, vitamin D, iron, zinc, and essential amino acids is maintained, particularly in older adults at increased risk of sarcopenia. This perspective supports the concept that dietary interventions should be interpreted within an integrated nutritional framework rather than as isolated nutrient exposures.

Maintenance of appropriate energy balance also represents a critical determinant of healthy aging trajectories. Chronic positive energy balance promotes visceral adipose tissue accumulation, ectopic lipid deposition, insulin resistance, and amplification of low-grade inflammatory activity [141]. Conversely, inadequate caloric and protein intake may accelerate anabolic resistance, impair muscle protein synthesis, and exacerbate sarcopenia-related functional decline [142]. Adequate protein intake therefore becomes particularly important during aging, although optimal requirements likely vary according to physical activity level, metabolic health, anabolic sensitivity, and comorbidity burden.

Importantly, dietary effects do not occur independently but interact closely with other lifestyle-related factors, especially physical activity and exercise adaptation [143]. Emerging evidence suggests that combined lifestyle interventions may exert complementary or potentially synergistic effects through coordinated modulation of inflammatory, mitochondrial, metabolic, and anabolic pathways. Consequently, nutritional strategies targeting inflammaging and sarcopenia should increasingly be considered within integrated multi-component lifestyle approaches rather than as isolated therapeutic interventions.

### 5.3. Sleep, Circadian Rhythms, and Stress

Sleep quality, circadian regulation, and psychological stress are increasingly recognized as important, yet frequently underappreciated, modulators of inflammaging and sarcopenia [144]. These factors influence multiple neuroendocrine, metabolic, inflammatory, and anabolic pathways that collectively shape healthy aging trajectories.

Sleep disturbances and circadian misalignment have been consistently associated with increased systemic inflammatory activity, impaired metabolic regulation, and reduced physiological resilience [145]. Both short sleep duration and poor sleep quality are linked to elevated circulating levels of pro-inflammatory cytokines, including IL-6 and TNF-α, alongside impairments in muscle protein synthesis and recovery processes [146]. In parallel, disruption of circadian rhythmicity alters the temporal regulation of key endocrine systems, including cortisol, melatonin, growth hormone, and insulin signaling, thereby influencing energy metabolism, mitochondrial function, and anabolic homeostasis [147].

At the molecular level, circadian regulation is closely integrated with inflammatory and metabolic signaling pathways. Dysregulation of circadian clock mechanisms may amplify oxidative stress, impair mitochondrial adaptation, and disrupt cellular repair processes, thereby contributing to inflammaging and age-related functional decline.

Chronic psychological stress further promotes a catabolic and pro-inflammatory physiological state, primarily through sustained activation of the hypothalamic–pituitary–adrenal (HPA) axis and prolonged glucocorticoid exposure [148]. Persistently elevated cortisol levels stimulate muscle protein breakdown, impair anabolic signaling, exacerbate insulin resistance, and contribute to chronic low-grade inflammatory activation [149]. Together, these alterations may accelerate skeletal muscle deterioration and broader metabolic dysfunction during aging.

Importantly, sleep quality, circadian alignment, and stress-related factors are potentially modifiable through behavioral and lifestyle-based interventions. Emerging evidence suggests that optimizing sleep patterns, restoring circadian synchronization, and implementing integrated stress-management strategies may beneficially influence inflammatory regulation, metabolic homeostasis, and physical function in older adults. Collectively, these observations support the concept that healthy aging is shaped not only by nutritional and physical activity-related factors but also by broader behavioral and chronobiological processes. Consequently, sleep- and stress-targeted interventions should increasingly be incorporated into integrated multi-domain strategies aimed at mitigating inflammaging, sarcopenia, and age-related functional decline.

## 6. Potential Interactions Between Bioactive Compounds and Lifestyle Factors

### 6.1. Interactions Between Nutrition and Physical Activity

The interactions between bioactive compounds and physical activity have emerged as a highly relevant area in healthy aging research, particularly in the context of inflammaging, metabolic resilience, and skeletal muscle preservation [19,76,118,130]. Evidence from mechanistic and human studies suggests that the effects of these interventions extend beyond simple additive models and involve context-dependent interactions mediated through adaptive stress responses, metabolic regulation, and redox-sensitive signaling pathways [23,70,93,124,150].

A central framework underlying these interactions is hormesis, whereby exposure to low or moderate physiological stressors induces cellular adaptations that enhance resilience and functional capacity [23,70,150]. Plant-derived bioactive compounds, including polyphenols, flavonoids, and certain lipid-derived mediators, appear capable of modulating exercise-induced signaling pathways rather than acting solely as direct antioxidants [19,48,68,76]. Mechanistic evidence suggests that moderate activation of redox-sensitive pathways, including Nrf2–Keap1 signaling, supports antioxidant defense, mitochondrial function, and cellular stress adaptation through tightly regulated redox signaling [23,70,71,124,150].

Exercise-induced Reactive Oxygen Species (ROS) and Reactive Nitrogen Species (RNS) are increasingly recognized as signaling mediators involved in mitochondrial biogenesis, metabolic adaptation, inflammatory regulation, and skeletal muscle remodeling through pathways such as NF-κB, AMPK, and Nrf2 [23,124,125,150].

Physical activity may also enhance responsiveness to nutritional bioactives by increasing tissue perfusion, metabolic turnover, membrane transport activity, and intracellular signaling efficiency. In parallel, skeletal muscle-derived myokines and extracellular vesicles may facilitate communication between exercise adaptation, immune regulation, and metabolic homeostasis. Importantly, these interactions appear highly dependent on dose, timing, physiological context, and baseline metabolic status. Human intervention studies indicate that excessive antioxidant supplementation may blunt exercise-induced mitochondrial biogenesis and endogenous antioxidant adaptation, highlighting the importance of balanced rather than maximal suppression of oxidative stress [150,151,152,153].

Overall, current evidence supports integrated lifestyle-based approaches rather than reductionist single-factor interventions. Observational studies and selected intervention trials suggest that coordinated combinations of dietary patterns, bioactive compounds, and exercise may contribute to the mitigation of inflammaging, preservation of skeletal muscle function, and promotion of healthy aging trajectories, particularly when tailored to individual physiological and metabolic characteristics.

### 6.2. Integrated Regulation of Inflammation, Oxidative Stress, and Muscle Metabolism

The biological mechanisms underlying inflammaging and sarcopenia do not operate as isolated processes but rather as components of an interconnected network integrating inflammatory signaling, redox homeostasis, mitochondrial function, and energy metabolism [25,34,41]. Within this framework, chronic low-grade inflammation, oxidative stress, impaired proteostasis, and metabolic dysfunction interact dynamically to drive age-related functional decline.

Redox homeostasis represents a central regulatory component of this network. According to the concept of hormesis, moderate levels of oxidative, metabolic, or mechanical stress can induce adaptive responses that enhance cellular resilience and metabolic flexibility [151,152]. Conversely, excessive stress exposure or insufficient adaptive stimulation may promote mitochondrial dysfunction, impaired repair capacity, and persistent inflammatory activation [151,153].

Mechanistic studies indicate that bioactive compounds interact with these regulatory systems at multiple molecular levels. Polyphenols, carotenoids, and lipid-derived bioactive compounds modulate redox-sensitive signaling pathways and influence the expression of antioxidant enzymes, detoxification pathways, and inflammatory mediators [48,68,84,93]. In parallel, these compounds may attenuate chronic inflammatory signaling through modulation of cytokine production and innate immune activation [7,49,61,101].

Exercise and bioactive compounds appear to influence several common signaling hubs involved in cellular adaptation and metabolic regulation, including Nrf2, NF-κB, HIF-1α, AMPK, and mTOR pathways [43,70,124,151]. Regulation of these pathways affects antioxidant defense, mitochondrial function, inflammatory activity, substrate utilization, and anabolic responsiveness.

**Figure 1:**
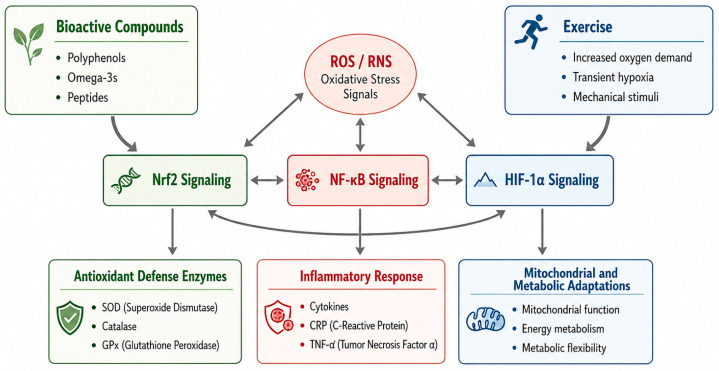
Conceptual schematic framework illustrating hypothesized and partially evidence-supported interactions among exercise-induced signals, bioactive compounds, redox-sensitive signaling pathways, and downstream adaptive responses involved in healthy aging and metabolic resilience. The figure is intended for conceptual interpretation and does not imply direct causal relationships for all depicted interactions. Abbreviations: ROS/RNS, reactive oxygen and nitrogen species; NF-κB, nuclear factor kappa B; Nrf2, nuclear factor erythroid 2-related factor 2; HIF-1α, hypoxia-inducible factor 1-alpha; SOD, superoxide dismutase; GPx, glutathione peroxidase; CRP, C-reactive protein; TNF-α, tumor necrosis factor alpha. ↔ bidirectional interaction; → directional regulation.

As illustrated in Figure 1, bioactive compounds, ROS/RNS, oxygen availability, mitochondrial activity, and exercise-related stimuli interact within an integrated regulatory network influencing Nrf2-, NF-κB-, and HIF-1α-dependent signaling pathways [150,151,152,153]. These interactions contribute to the coordinated regulation of antioxidant defenses, inflammatory mediators, stress-response proteins, and metabolic regulators at both cellular and systemic levels [19,76,124]. Overall, inflammaging and sarcopenia appear to arise from dysregulation of interconnected adaptive mechanisms rather than single pathways, suggesting that effective interventions will likely require coordinated multi-target approaches aimed at preserving metabolic resilience and functional homeostasis.

The integrated regulation of these pathways gives rise to several clinically relevant adaptations, including improved mitochondrial function and bioenergetic efficiency, attenuation of chronic low-grade inflammatory activity, optimization of muscle protein turnover, enhanced metabolic flexibility, and increased cellular resilience [23,41,124,129,151]. Importantly, these adaptive responses are inherently non-linear and highly context-dependent. Excessive antioxidant exposure or inadequately periodized exercise interventions may disrupt physiological redox signaling, potentially leading to reductive stress, impaired mitochondrial adaptation, and attenuation of endogenous stress-response mechanisms [150,153]. These observations emphasize that optimal physiological adaptation depends not on maximal suppression of oxidative processes but rather on the maintenance of a finely regulated redox environment capable of supporting adaptive signaling and metabolic homeostasis [151,153].

**Figure 2:**
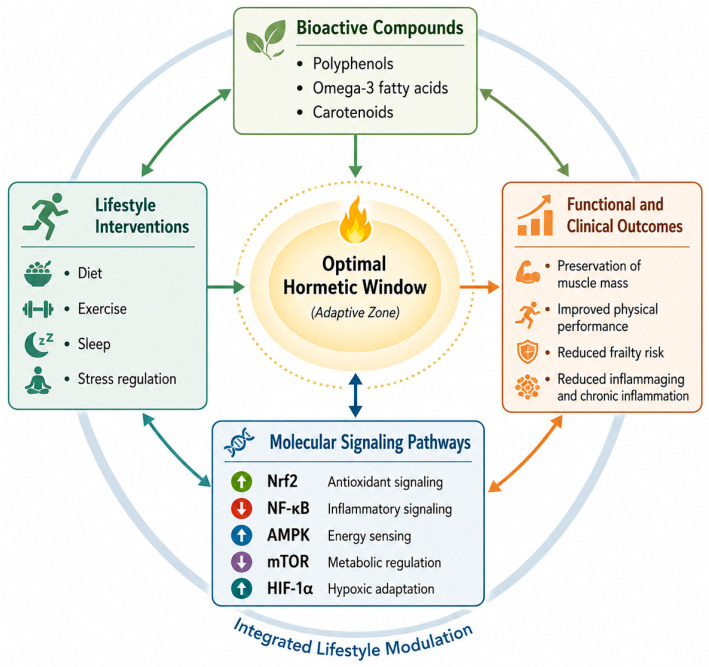
Conceptual schematic framework illustrating hypothesized and partially evidence-supported interactions among lifestyle interventions, bioactive compounds, molecular signaling pathways, and functional outcomes in healthy aging. The figure is intended for conceptual interpretation and does not imply direct causal relationships for all depicted interactions. Abbreviations: NF-κB, nuclear factor kappa B; Nrf2, nuclear factor erythroid 2-related factor 2; mTOR, mechanistic target of rapamycin; AMPK, AMP-activated protein kinase; HIF-1α, hypoxia-inducible factor 1-alpha. ↑, increase/activation; ↓, decrease/inhibition; ↔, bidirectional interaction. Arrows indicate putative regulatory interactions and adaptive signaling relationships among lifestyle interventions, bioactive compounds, molecular pathways, and functional outcomes. Colors are used solely to distinguish the major conceptual domains of the framework and do not imply specific biological effects or quantitative relationships.

As illustrated in Figure 2, inflammaging and sarcopenia can be conceptualized within a multi-layered integrative framework in which lifestyle factors and bioactive compounds may exert complementary and partially convergent effects across interconnected molecular, metabolic, inflammatory, mitochondrial, and neuromuscular pathways [19,25,32,34]. Rather than arising from a single dominant mechanism, these age-related processes emerge from dynamic interactions within complex adaptive biological networks, where the magnitude and direction of responses are strongly influenced by dose, timing, physiological reserve, and environmental context [1,152,153].

Within this framework, exercise, dietary patterns, sleep regulation, stress modulation, and nutritional bioactives influence key signaling hubs involved in cellular adaptation, including Nrf2, NF-κB, AMPK, mTOR, mitochondrial quality control systems, and immune-metabolic pathways [43,70,124,129,151]. Observational studies and randomized controlled trials have reported greater functional benefits with integrated lifestyle interventions than with some isolated single-target approaches, although causal mechanistic links remain incompletely understood [9,118,130].

Overall, the interactions between bioactive compounds and lifestyle-related factors may be viewed as a dynamic adaptive system requiring individualized modulation rather than standardized intervention paradigms [10,152]. Emerging evidence regarding antioxidant supplementation, oxidative stress signaling, Nrf2-mediated adaptive responses, exercise hormesis, and mitohormetic mechanisms further supports the concept that optimal adaptation depends on context-specific biological responses rather than uniform interventions [153,154,155,156,157,158]. This perspective closely aligns with emerging concepts in precision lifestyle medicine, systems biology, and geroscience, and may provide a conceptual foundation for the development of personalized multi-component strategies aimed at preserving functional capacity and promoting healthy aging trajectories [2,9,25,159,160,161,162,163,164,165].

## 7. Human Evidence and Clinical Relevance

### 7.1. Observational Epidemiological Studies

Available epidemiological evidence consistently supports a close and biologically plausible association between dietary patterns, lifestyle-related factors, chronic low-grade inflammation, and age-related declines in skeletal muscle function and physical performance [163,166]. Observational studies and meta-analyses increasingly suggest that long-term lifestyle behaviors substantially influence trajectories of inflammaging and sarcopenia through interconnected inflammatory, metabolic, anabolic, redox-regulatory, and adaptive stress-response pathways [163,166].

Several meta-analyses have demonstrated that lower Dietary Inflammatory Index (DII) scores, reflecting more anti-inflammatory dietary patterns, are associated with greater muscle mass, higher muscle strength, and a reduced risk of sarcopenia in older adults [167,168,169,170]. These findings support the concept that dietary inflammatory potential may represent an important modifiable determinant of muscle health and functional aging. Emerging evidence further suggests that bioactive dietary compounds, including polyphenol-rich foods, may contribute to the preservation of muscle function through antioxidant, redox-regulatory, Nrf2-mediated, metabolic, and epigenetic mechanisms, thereby supporting healthy aging and geroscience-oriented intervention strategies [161,164,165].

Similarly, higher adherence to the Mediterranean diet has been consistently associated with more favorable body composition, improved gait speed, better physical performance, and lower frailty prevalence across diverse observational populations [168,171,172]. Importantly, the beneficial effects of Mediterranean-style dietary patterns likely arise from the combined influence of multiple nutritional and behavioral factors, including higher intake of polyphenols, unsaturated fatty acids, dietary fiber, and antioxidant-rich foods, together with broader cardiometabolic benefits.

Nevertheless, these associations are not uniformly reflected across all diagnostic definitions of sarcopenia or frailty. Considerable heterogeneity exists between studies with respect to sarcopenia criteria, body composition assessment methods, physical performance measurements, and participant characteristics [173,174]. Such variability complicates direct comparison across cohorts and suggests that structural and functional outcomes may differ in their responsiveness and sensitivity to dietary influences.

The relationship between inflammatory biomarkers and functional decline is further supported by meta-analytic evidence demonstrating that elevated circulating levels of IL-6, TNF-α, and CRP are consistently associated with lower muscle strength, reduced muscle mass, impaired mobility, and increased frailty risk [175,176,177]. Although these findings are predominantly associative and cannot establish causality, they strongly reinforce the biological plausibility of inflammaging as a clinically relevant contributor to functional decline and adverse aging trajectories [178]. Collectively, epidemiological evidence supports the view that lifestyle-related exposures—including dietary quality, physical activity, sleep patterns, and broader behavioral factors—interact across the lifespan to influence inflammatory burden, metabolic resilience, and skeletal muscle health. These observations provide an important translational foundation for the development of integrated preventive strategies targeting healthy aging and functional preservation.

### 7.2. Randomized Controlled Dietary and Lifestyle Interventions

Meta-analyses of randomized controlled trials provide increasing evidence that dietary and lifestyle interventions may exert not only associative but also causal effects on key biological mechanisms implicated in inflammaging and sarcopenia [163,179,180,181]. Collectively, these findings support the concept that modifiable lifestyle-related exposures can influence inflammatory regulation, metabolic adaptation, and functional aging trajectories in clinically meaningful ways.

Meta-analyses investigating Mediterranean dietary interventions have demonstrated significant reductions in circulating inflammatory biomarkers, particularly CRP and IL-6 [182,183]. In several analyses, the observed effects corresponded to measurable standardized effect sizes, suggesting that the anti-inflammatory benefits may extend beyond statistical significance to potential clinical relevance [184,185]. Nevertheless, the magnitude of these effects is generally moderate, indicating that the biological impact of dietary interventions is likely cumulative and dependent on long-term adherence, baseline metabolic status, and broader lifestyle context.

Evidence from randomized trials and meta-analyses evaluating omega-3 fatty acid supplementation indicates that these interventions primarily improve functional rather than structural outcomes [186]. In older adults, supplementation has been associated with significant improvements in lower limb muscle strength, gait-related performance, and functional mobility assessments, including the Timed Up and Go and sit-to-stand tests [187,188,189]. By contrast, effects on skeletal muscle mass are generally smaller and less consistent across studies, supporting the notion that improvements in muscle quality, neuromuscular efficiency, and metabolic function may occur independently of substantial changes in muscle size alone [187].

Among currently available non-pharmacological interventions, physical activity—particularly resistance training—demonstrates the strongest and most reproducible evidence base. Meta-analyses consistently report significant improvements in muscle strength, physical performance, mobility, and selected inflammatory biomarkers following structured exercise interventions [190,191,192,193]. Importantly, exercise exerts broad multi-system effects involving inflammatory modulation, mitochondrial adaptation, anabolic signaling, and improvements in metabolic homeostasis.

These findings suggest that exercise should not be viewed merely as an adjunctive intervention but rather as a central therapeutic component in strategies targeting inflammaging and sarcopenia [194,195]. From a geroscience perspective, physical activity may represent one of the most effective currently available interventions capable of simultaneously modulating multiple hallmarks of biological aging and functional decline.

Despite these encouraging findings, important limitations remain across intervention studies, including heterogeneity in participant characteristics, intervention duration, training protocols, dietary composition, and outcome assessment methodologies. Future large-scale, long-term trials integrating mechanistic biomarkers with clinically relevant functional endpoints will be essential to clarify optimal intervention strategies and identify populations most likely to benefit from combined lifestyle-based approaches.

### 7.3. Functional Outcomes: Muscle Strength, Physical Performance, Frailty, and Quality of Life

Functional outcomes are of particular clinical importance because they directly reflect the integrated physiological consequences of age-related biological processes and frequently predict morbidity, disability, hospitalization, and mortality more accurately than isolated molecular biomarkers alone. In the context of healthy aging, preservation of physical function and independence represents a primary therapeutic objective rather than merely a secondary consequence of improved biological parameters.

Meta-analytic evidence consistently indicates that anti-inflammatory dietary patterns and regular physical activity exert beneficial effects on muscle strength, gait speed, functional mobility, and overall physical performance, including validated assessments such as the Short Physical Performance Battery (SPPB) and the Timed Up and Go (TUG) test [173,190,192,196]. Importantly, these benefits have been observed across diverse populations and intervention settings, supporting their translational and clinical relevance.

Resistance training and multicomponent exercise interventions appear particularly effective in improving lower extremity strength, balance, mobility, and frailty-related outcomes. In parallel, adherence to Mediterranean-style and other anti-inflammatory dietary patterns has been associated with better preservation of functional capacity and lower frailty prevalence in aging populations. These observations further support the concept that nutritional and exercise-related interventions exert complementary and potentially synergistic effects on functional aging trajectories. A particularly important finding emerging from epidemiological and meta-analytic evidence is the close and frequently dose-dependent association between elevated inflammatory biomarkers and functional decline. Higher circulating levels of IL-6, TNF-α, and CRP are consistently associated with slower gait speed, reduced muscle strength, impaired mobility, and increased frailty risk [175,176,177]. These relationships reinforce the concept that inflammaging is not merely a laboratory-defined phenomenon but a clinically manifest biological process directly linked to deterioration in physical function and loss of independence.

Importantly, functional decline itself may further amplify inflammatory and metabolic dysregulation through reduced physical activity, impaired mitochondrial adaptation, anabolic resistance, and increased adiposity, thereby contributing to a self-reinforcing cycle of frailty progression. This bidirectional interaction highlights the importance of early preventive strategies aimed at preserving functional reserve before irreversible decline becomes established. Collectively, current evidence supports the integration of functional performance measures alongside molecular and inflammatory biomarkers when evaluating interventions targeting inflammaging and sarcopenia. Such an approach may provide a more clinically meaningful assessment of healthy aging outcomes and facilitate translation of mechanistic findings into practical lifestyle-based therapeutic strategies (Table 2).

### 7.4. Clinical Applicability and Limitations

Overall, current human evidence supports the concept that bioactive compound-rich dietary patterns and lifestyle-based interventions exert clinically meaningful effects on key mechanisms underlying inflammaging and sarcopenia [231,232,233]. Nevertheless, several important limitations should be considered when translating these findings into clinical practice.

A major challenge is the substantial heterogeneity of the available evidence, particularly regarding study populations, intervention protocols, dosages, treatment duration, and outcome assessment methods. Such variability complicates the formulation of standardized clinical recommendations and reflects both the biological complexity of aging-related processes and methodological differences across studies [231,234].

Another critical consideration is the pronounced interindividual variability in responsiveness to nutritional and lifestyle interventions. Factors including genetic background, biological age, baseline nutritional status, comorbidities, physical activity level, and gut microbiome composition may substantially influence bioavailability and therapeutic efficacy [29,235,236]. This issue is especially relevant in older populations, in whom reduced physiological reserve and multimorbidity may alter adaptive capacity and intervention responsiveness.

Importantly, although numerous short-term randomized controlled trials have reported favorable effects on inflammatory markers, muscle function, and physical performance, large-scale and long-duration intervention studies remain relatively limited [173,231,232]. Consequently, the long-term sustainability, safety, and clinical magnitude of these effects remain incompletely established.

In addition, many currently available studies evaluate isolated interventions despite the fact that inflammaging and sarcopenia are fundamentally multifactorial and network-driven processes. This highlights the need for integrative study designs capable of assessing combined and potentially synergistic effects of nutrition, physical activity, sleep, stress regulation, and metabolic factors within real-world clinical settings. Overall, the current body of evidence strongly supports the role of dietary and lifestyle interventions as promising multi-target strategies for the modulation of inflammaging and sarcopenia. However, future research should prioritize standardized methodologies, mechanistically informed biomarkers, longer follow-up periods, and precision-based intervention models to improve clinical applicability and translational relevance.

## 8. Sustainability and Future Directions

The growing burden of age-related chronic diseases represents not only a biomedical challenge but also a societal and sustainability concern, highlighting the need for coordinated nutritional and public health strategies. In this context, dietary patterns rich in natural bioactive compounds, particularly plant-forward dietary models, may simultaneously target mechanisms implicated in inflammaging and sarcopenia while exerting a lower environmental impact.

The Mediterranean diet and related plant-based dietary patterns are generally associated with lower greenhouse gas emissions, reduced resource use, and greater food system sustainability. At the same time, they provide abundant anti-inflammatory and antioxidant bioactive compounds, linking healthy aging with environmental sustainability.

However, implementation of sustainable dietary strategies in older adults requires careful consideration. Aging is frequently accompanied by anabolic resistance, reduced appetite, impaired nutrient absorption, multimorbidity, and declining functional capacity, increasing the risk of inadequate protein and micronutrient intake. Sustainable dietary patterns may contribute to healthy aging when adequate protein, vitamin B12, vitamin D, iron, zinc, and essential amino acid intake is maintained, particularly in individuals at risk of sarcopenia.

Future research should focus on personalized, multi-component approaches integrating nutritional, metabolic, microbiome-related, and lifestyle factors. Advanced omics technologies, digital health tools, and artificial intelligence-based analytics may help identify responder phenotypes and optimize intervention strategies. Overall, sustainable lifestyle approaches may contribute to preserving physical function and promoting healthy aging while reducing the societal burden of age-related decline.

### Practical Translational Perspective

Current evidence suggests that beneficial non-pharmacological approaches targeting inflammaging and sarcopenia are unlikely to depend on isolated interventions alone, but rather on multicomponent lifestyle-based approaches. In clinical practice, this framework may include adherence to an anti-inflammatory dietary pattern—particularly a Mediterranean-style diet—combined with adequate protein intake, regular resistance and aerobic exercise, optimization of sleep quality and circadian alignment, and selective incorporation of bioactive compounds such as omega-3 fatty acids.

To enhance translational applicability, Table 3 summarizes selected lifestyle- and nutrition-based strategies with the most consistent available clinical and mechanistic evidence for targeting inflammaging and sarcopenia. Rather than representing formal therapeutic guidelines, these approaches should be interpreted as evidence-informed practical frameworks integrating dietary, exercise-related, metabolic, and behavioral factors relevant to healthy aging trajectories.

Among currently available strategies, the combination of a Mediterranean-style dietary pattern, adequate protein intake, regular resistance and aerobic exercise, and selected omega-3 supplementation may represent one of the most consistently supported multimodal approaches for preserving muscle function and potentially mitigating inflammaging in older adults [55,80,118,123]. However, intervention effects appear to differ substantially across sarcopenia-related outcomes. Improvements in inflammatory biomarkers (e.g., CRP, IL-6, TNF-α) and functional measures such as muscle strength and physical performance are among the most consistently reported findings, whereas effects on muscle mass are generally smaller and more heterogeneous across studies. These observations highlight the importance of evaluating muscle quantity, muscle function, inflammatory status, frailty-related outcomes, and quality of life as distinct but complementary domains.

Importantly, substantial interindividual variability exists in responsiveness to these interventions, influenced by factors including age, metabolic status, physical activity level, comorbidities, medication use, and gut microbiome composition. Consequently, future preventive and therapeutic approaches will likely increasingly emphasize tailored lifestyle interventions aimed at preserving functional capacity and metabolic resilience during aging. These considerations should be interpreted within the context of individual clinical status. Factors including multimorbidity, frailty severity, renal function, polypharmacy, anticoagulant therapy, baseline nutritional status, and adherence-related barriers may substantially influence the safety, feasibility, and effectiveness of lifestyle-based interventions in older adults. Consequently, individualized assessment remains essential when translating these strategies into clinical practice.

## 9. Limitations and Research Gaps

Despite the rapidly expanding body of evidence, several important methodological and translational limitations continue to constrain interpretation and clinical applicability.

One of the major challenges is the substantial heterogeneity of the available literature. Differences in study populations, biological age, nutritional status, intervention protocols, dosages, treatment duration, and outcome assessment methods complicate direct comparisons between studies and limit the development of standardized recommendations. Considerable variability also exists in the operational definitions and diagnostic criteria used for sarcopenia, frailty, and inflammatory status.

Another unresolved issue involves characterization of dose–response relationships and optimal intervention thresholds. The biological effects of many bioactive compounds are highly context-dependent and frequently non-linear, being influenced by bioavailability, metabolism, timing of administration, and baseline physiological status. This complexity is particularly relevant within the framework of hormesis, where both insufficient and excessive exposure may produce suboptimal or maladaptive effects.

In addition, relatively few large-scale and long-duration randomized controlled trials have evaluated integrated lifestyle interventions. Although short-term studies generally support favorable effects on inflammatory regulation, muscle function, and physical performance, evidence regarding long-term sustainability and clinically meaningful outcomes remains limited.

Most currently available studies continue to investigate isolated interventions despite the multifactorial nature of biological aging. Interactions among nutrition, physical activity, sleep, circadian regulation, psychosocial stress, and metabolic health remain insufficiently characterized, and the extent to which observed effects reflect true biological synergy remains incompletely understood.

Future research should therefore prioritize standardized methodologies, harmonized outcome measures, longitudinal study designs, and integrated intervention models combining molecular, functional, and clinical endpoints. Addressing these limitations may be important for improving the translational relevance of lifestyle- and bioactive compound-based strategies targeting inflammaging and sarcopenia.

## 10. Conclusions

Inflammaging and sarcopenia should be regarded as interconnected components of a shared pathophysiological network involving chronic inflammation, redox dysregulation, mitochondrial dysfunction, impaired anabolic signaling, and progressive decline in muscle function. Current evidence suggests that meaningful modulation of these processes is unlikely to be achieved through single-target interventions alone.

Natural bioactive compounds and lifestyle-related factors exert coordinated effects on multiple hallmarks of aging through interconnected inflammatory, metabolic, and redox-sensitive pathways. Importantly, these effects appear to operate within a hormetic framework in which adaptive responses are strongly influenced by biological context, dose, timing, and physiological status.

The available evidence supports consideration of integrated lifestyle-based strategies incorporating dietary patterns rich in natural bioactive compounds, regular physical activity, adequate sleep, and circadian health. However, substantial interindividual variability in responsiveness underscores the need for individualized assessment when translating these interventions into practice. Overall, multi-target lifestyle and nutritional interventions may represent promising approaches for preserving physical function, reducing frailty, and promoting healthy aging trajectories.

## Figures and Tables

**Table 1:** Bioactive compounds, principal molecular targets, and current level of evidence in inflammaging and sarcopenia.

Compound Class	Principal Molecular Targets	Effects on Inflammaging	Effects on Sarcopenia	Human Clinical Evidence	Preclinical/ Mechanistic Evidence
Polyphenols and flavonoids [19,50,51,52,53]	NF-κB ↓; AMPK ↑; SIRT1 ↑; Nrf2 ↑	Reduced inflammatory markers (CRP, IL-6); improved redox homeostasis and mitochondrial adaptation	Limited and heterogeneous effects on muscle mass; potential improvements in mitochondrial function and metabolic resilience	Moderate	Strong
Omega-3 fatty acids [54,55,56,57,58]	NF-κB ↓; PPAR ↑; resolvin pathways ↑	Reduced inflammatory signaling; enhanced resolution of inflammation	Improved muscle strength and physical performance; inconsistent effects on muscle mass	Moderate–Strong	Strong
Carotenoids [59,60,61,62,63]	ROS ↓; NF-κB ↓; antioxidant pathways ↑	Reduced oxidative stress and modest anti-inflammatory effects	Predominantly observational evidence regarding muscle-related outcomes	Limited	Moderate
Bioactive peptides [64,65,66,67]	mTOR ↑; MPS ↑	Limited evidence	Potential stimulation of muscle protein synthesis and anabolic signaling, primarily in preclinical models	Limited	Emerging

Evidence grades are descriptive categories assigned by the authors based on the consistency, quantity, and methodological quality of currently available human and preclinical studies and do not represent a formal GRADE assessment or other standardized evidence-rating system. Evidence categories were defined as follows: Strong = consistent evidence from multiple randomized controlled trials and meta-analyses; Moderate = evidence supported by human studies but with some inconsistency or heterogeneity; Limited = evidence derived primarily from observational studies or small clinical studies; Emerging = evidence based predominantly on preclinical investigations. Abbreviations: NF-κB, nuclear factor kappa B; AMPK, AMP-activated protein kinase; PPAR, peroxisome proliferator-activated receptor; SIRT1, sirtuin 1; Nrf2, nuclear factor erythroid 2–related factor 2; mTOR, mechanistic target of rapamycin; ROS, reactive oxygen species; MPS, muscle protein synthesis; CRP, C-reactive protein; IL-6, interleukin-6; ↑ Increase; ↓ decrease.

**Table 2:** Summary of meta-analytic evidence from human studies on lifestyle interventions targeting inflammaging, sarcopenia, physical function, and frailty in older adults.

Intervention	Primary Outcomes	Overall Effect	Consistency of Evidence	Key Findings	References
Mediterranean diet	CRP, IL-6, frailty, physical performance	↓ Inflammation; ↓ frailty risk	High	Consistently associated with lower systemic inflammation, reduced frailty prevalence, and improved physical performance in older adults. Moderate but reproducible pooled effect sizes across studies.	[197,198,199,200,201,202,203,204]
Omega-3 fatty acids (*n*-3 PUFA)	Muscle strength, gait speed, muscle performance	↑ Muscle strength and function	Moderate	Most meta-analyses demonstrate improvements in handgrip strength and physical performance, whereas effects on muscle mass remain less consistent and more heterogeneous.	[186,205,206,207,208,209]
Aerobic exercise	CRP, IL-6, TNF-α, gait speed, SPPB	↓ Inflammation; ↑ physical performance	Very high	Strong evidence supports reductions in chronic low-grade inflammation together with improvements in gait speed, endurance, and functional mobility.	[210,211,212,213,214]
Resistance training	Muscle strength, lean mass, inflammatory markers	↑↑ Strength; ↓ inflammatory markers	Very high	Resistance exercise demonstrates the most robust and reproducible improvements in muscle strength, functional capacity, and selected inflammatory biomarkers.	[215,216,217,218,219]
Multicomponent exercise programs	Frailty, TUG, SPPB, quality of life	↑↑ Functional outcomes; ↓ frailty	Very high	Combined aerobic, resistance, balance, and functional interventions consistently improve physical performance, mobility, and frailty status in older adults.	[215,220,221,222,223,224,225,226]
Nutrition plus exercise interventions	Muscle mass, strength, sarcopenia outcomes	↑ Physical function	High	Combined interventions generally outperform isolated nutritional or exercise approaches for sarcopenia-related functional outcomes.	[222,227,228,229]
Inflammation–frailty association	IL-6, CRP, TNF-α vs. frailty and muscle function	Positive association between inflammation and decline	High	Elevated inflammatory biomarkers are consistently associated with reduced muscle strength, slower gait speed, increased frailty, and adverse aging outcomes.	[202,219,230]

Abbreviations: CRP, C-reactive protein; IL-6, interleukin-6; TNF-α, tumor necrosis factor alpha; SPPB, Short Physical Performance Battery; TUG, Timed Up and Go; PUFA, polyunsaturated fatty acids. ↑ moderate increase; ↑↑ pronounced increase; ↓ decrease.

**Table 3:** Representative lifestyle and nutritional strategies associated with favorable outcomes in inflammaging and sarcopenia.

Intervention	Representative Range Used in Human Studies	Main Target/Mechanism	Representative Evidence
Mediterranean-style diet	Long-term adherence to a Mediterranean-style dietary pattern	Inflammatory regulation, oxidative stress modulation, metabolic resilience	[80,137,182,183,201,202,203,204]
Protein intake	0.8–1.2 g/kg/day (up to 1.5 g/kg/day in active or catabolic older adults)	Muscle protein synthesis, anabolic support	[31,64,228,236]
Resistance training	2–3 sessions/week involving major muscle groups	Muscle strength, anabolic signaling, neuromuscular adaptation	[123,190,191,192,193,216,217,218]
Aerobic exercise	≥150 min/week moderate-intensity activity	Metabolic and inflammatory regulation, mitochondrial adaptation	[118,210,211,212,213,214]
Omega-3 fatty acids	250 mg–3 g/day combined EPA + DHA supplementation	Inflammation resolution, muscle metabolism	[55,56,57,205,206,207,208,209]
Polyphenol-rich foods	Habitual intake of polyphenol-rich plant foods	Redox signaling, endothelial and mitochondrial function	[50,51,52,53,68,70,81]
Sleep optimization	~7–9 h/night	Inflammatory and endocrine regulation	[144,145,146]
Circadian alignment	Consistent sleep–wake timing	Metabolic and mitochondrial homeostasis	[11,144,147]

Source: Authors’ own compilation based on the cited literature.

## Data Availability

Data sharing is not applicable to this article as no new data were created or analyzed in this study.

## Supplementary Materials

The following supporting information can be downloaded at: https://www.mdpi.com/article/10.3390/nu18121920/s1, Figure S1: Flow diagram summarizing the literature identification, screening, eligibility assessment, and study selection process used for the present narrative review. The diagram is intended to improve methodological transparency and does not represent a formal PRISMA systematic review process. Table S1: Representative literature search strategies used for evidence identification. Table S2: Representative human studies evaluating bioactive compounds relevant to inflammaging and sarcopenia.

## Abbreviations

AMPK	AMP-activated protein kinase
ATP	adenosine triphosphate
CRP	C-reactive protein
DAMPs	danger-associated molecular patterns
DHA	docosahexaenoic acid
DII	Dietary Inflammatory Index
DNA	deoxyribonucleic acid
EPA	eicosapentaenoic acid
HIF-1α	hypoxia-inducible factor 1-alpha
HPA	hypothalamic–pituitary–adrenal
IL-1β	interleukin-1 beta
IL-6	interleukin-6
IL-18	interleukin-18
Keap1	Kelch-like ECH-associated protein 1
MAPK	mitogen-activated protein kinase
MPB	muscle protein breakdown
MPS	muscle protein synthesis
mtDNA	mitochondrial DNA
mTOR	mechanistic target of rapamycin
NF-κB	nuclear factor kappa B
NLRP3	NLR family pyrin domain containing 3
Nrf2	nuclear factor erythroid 2–related factor 2
PPAR	peroxisome proliferator-activated receptor
PUFAs	polyunsaturated fatty acids
RCT	randomized controlled trial
RNS	reactive nitrogen species
ROS	reactive oxygen species
SASP	senescence-associated secretory phenotype
SIRT1	sirtuin 1
TNF-α	tumor necrosis factor alpha
