# Supplementary material for: Inflammaging and Sarcopenia as Interconnected Hallmarks of Aging: Integrative Roles of Bioactive Compounds and Lifestyle Interventions
Source: Nutrients. 2026 Jun 13;18(12):1920. doi: 10.3390/nu18121920 (PMC13306084; doi:10.3390/nu18121920)
Supplement: Supplementary file 1 [file nutrients-18-01920-s001.zip › nutrients-4338016-supplementary.pdf]

Supplementary Table S1. Representative literature search strategies used for evidence identification.

Database	Representative search strategy
PubMed/MEDLINE	("inflammaging" OR "chronic low-grade inflammation" OR immunosenescence OR "cellular senescence") AND (sarcopenia OR frailty OR "muscle strength" OR "physical performance") AND (polyphenols OR flavonoids OR carotenoids OR "omega-3 fatty acids" OR nutraceuticals OR nutrition)
PubMed/MEDLINE	("inflammaging" OR "chronic low-grade inflammation" OR immunosenescence) AND (sarcopenia OR frailty) AND ("physical activity" OR exercise OR "resistance training" OR lifestyle)
Scopus	TITLE-ABS-KEY (inflammaging OR "chronic low-grade inflammation" OR immunosenescence OR senescence) AND TITLE-ABS-KEY (sarcopenia OR frailty OR "muscle function" OR "muscle strength") AND TITLE-ABS-KEY (polyphenols OR flavonoids OR carotenoids OR "omega-3 fatty acids" OR nutraceuticals OR antioxidants)
Scopus	TITLE-ABS-KEY (inflammaging OR immunosenescence) AND TITLE-ABS-KEY (sarcopenia OR frailty) AND TITLE-ABS-KEY ("physical activity" OR exercise OR "resistance training" OR "lifestyle intervention" OR "healthy aging")
Web of Science Core Collection	inflammaging OR "chronic low-grade inflammation" OR immunosenescence AND sarcopenia OR frailty AND polyphenols OR flavonoids OR carotenoids OR "omega-3 fatty acids" OR nutraceuticals
Web of Science Core Collection	inflammaging OR immunosenescence AND sarcopenia OR frailty AND exercise OR "physical activity" OR "resistance training" OR lifestyle OR "healthy aging"
Additional mechanistic searches	inflammaging AND sarcopenia AND (NF-κB OR AMPK OR mTOR OR Nrf2 OR oxidative stress OR mitochondrial dysfunction OR mitophagy OR hormesis)
Additional clinical searches	sarcopenia AND older adults AND (handgrip strength OR gait speed OR SPPB OR DXA OR BIA OR frailty OR CRP OR IL-6 OR TNF-α)
Integrated lifestyle searches	inflammaging AND sarcopenia AND (nutrition OR dietary patterns OR bioactive compounds) AND (exercise OR physical activity OR resistance training)

Search limits:

• Databases searched: PubMed/MEDLINE, Scopus, and Web of Science Core Collection

• Final search date: March 2026

• Publication period: January 2010–March 2026

• Language restriction: English

• Study types prioritized: randomized controlled trials, systematic reviews, meta-analyses, observational human studies, and translational mechanistic investigations

• Landmark studies published before 2010 were included when considered essential for conceptual background.

Supplementary Figure S1. Literature selection process.

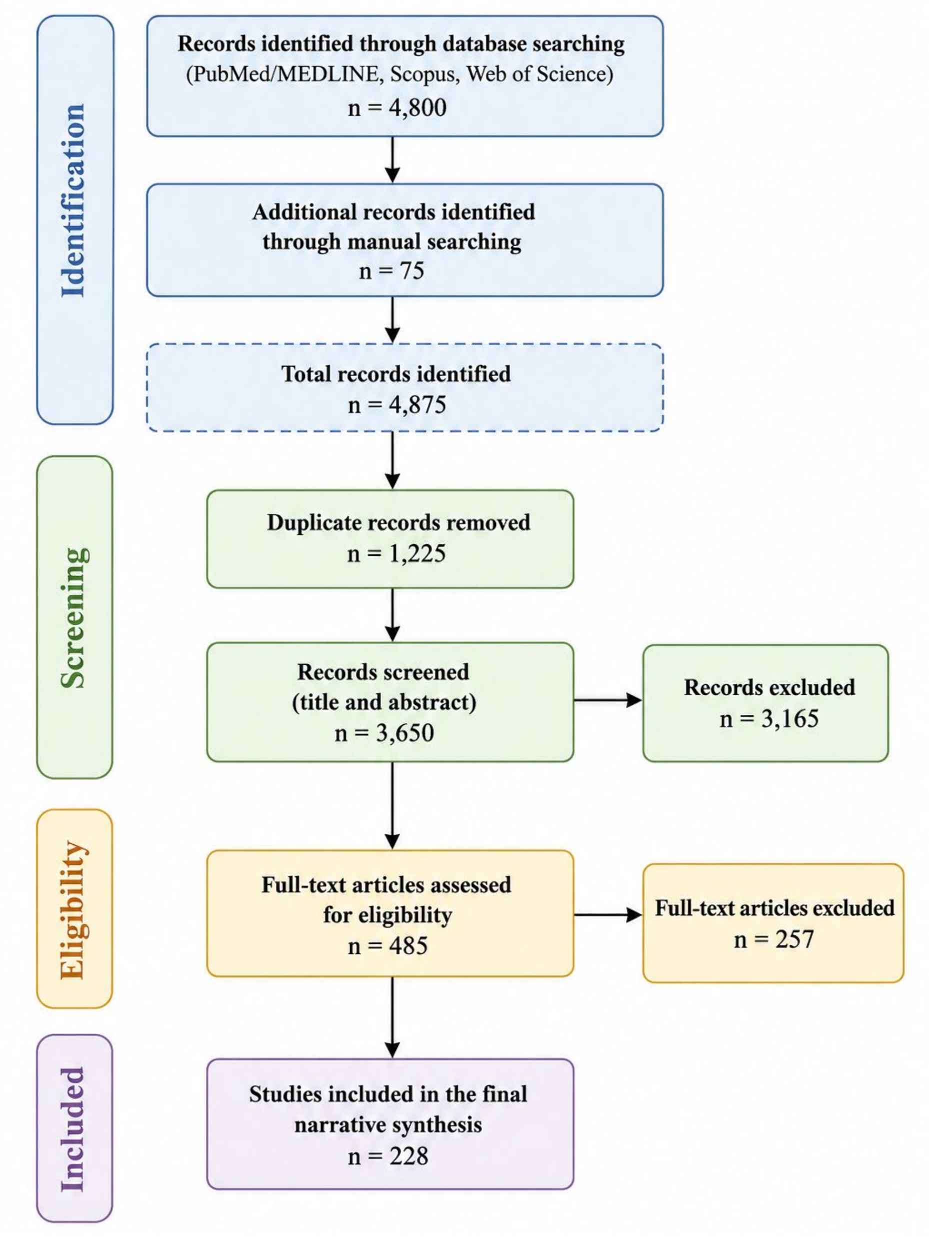

Figure S1. Flow diagram summarizing the literature identification, screening, eligibility assessment, and study selection process used for the present narrative review. The diagram is intended to improve methodological transparency and does not represent a formal PRISMA systematic review process.

Supplementary Table S2. Representative human studies evaluating bioactive compounds relevant to inflammaging and sarcopenia.

Compound class	Population	Formulation	Dose	Duration	Main outcomes	Representative human studies/reviews
Polyphenol-rich dietary patterns (Mediterranean diet-derived polyphenols)	Community-dwelling older adults	Polyphenol-rich dietary pattern	Habitual intake	6–24 months	Reduced CRP and IL-6, improved physical performance and metabolic health	[50,52,80,81,171,178,197,200]
Flavonoids (epicatechin, quercetin, catechin-containing preparations)	Adults with cardiovascular risk factors	Flavonoid supplementation (epicatechin-, quercetin-, or catechin-containing preparations)	500–1000 mg/day	8–24 weeks	Improved endothelial function, reduced oxidative stress and inflammation	[82,83]
Omega-3 fatty acids (EPA+DHA)	Older adults with or without sarcopenia risk	Fish oil supplementation	1–4 g/day EPA+DHA	3–12 months	Improved muscle strength and physical performance; inconsistent effects on muscle mass	[55–58,93,94,186,205–209]
Omega-3 fatty acids combined with exercise	Older adults	Fish oil capsules + exercise	2–3 g/day EPA+DHA	12–24 weeks	Enhanced anabolic responsiveness and functional performance in some studies	[55,57,205]

Carotenoids (lutein, lycopene, β-carotene)	Community-dwelling older adults	Dietary intake or supplementation	Variable	Variable	Lower oxidative stress and inflammation; possible associations with physical performance	[59–61,84,85,90,91]
Bioactive peptides (whey-derived peptides)	Older adults with reduced muscle function	Whey peptide supplementation	3–10 g/day peptides	8–24 weeks	Potential improvements in muscle protein synthesis and muscle function	[64–67,191]
Fermented food-derived bioactives	Older adults	Fermented dairy products and functional foods	Variable	4–24 weeks	Modulation of gut microbiota and inflammatory markers; limited evidence for sarcopenia outcomes	[109,114–117,132]

Source: Authors' compilation based on the cited literature. Abbreviations: CRP, C-reactive protein; IL-6, interleukin-6; EPA, eicosapentaenoic acid; DHA, docosahexaenoic acid; EGCG, epigallocatechin gallate. The table summarizes representative dose ranges, formulations, intervention durations, populations, and outcomes reported in human studies discussed in this review. Considerable heterogeneity exists across study designs, outcome measures, and intervention protocols; therefore, the values presented should be interpreted as illustrative rather than prescriptive.
